# Transmission routes of 2019-nCoV and controls in dental practice

**DOI:** 10.1038/s41368-020-0075-9

**Published:** 2020-03-03

**Authors:** Xian Peng, Xin Xu, Yuqing Li, Lei Cheng, Xuedong Zhou, Biao Ren

**Affiliations:** State Key Laboratory of Oral Diseases & National Clinical Research Center for Oral Diseases & Department of Cariology and Endodontics, West China Hospital of Stomatology, Sichuan University, Chengdu, China

**Keywords:** Policy and public health in microbiology, Risk factors

## Abstract

A novel β-coronavirus (2019-nCoV) caused severe and even fetal pneumonia explored in a seafood market of Wuhan city, Hubei province, China, and rapidly spread to other provinces of China and other countries. The 2019-nCoV was different from SARS-CoV, but shared the same host receptor the human angiotensin-converting enzyme 2 (ACE2). The natural host of 2019-nCoV may be the bat *Rhinolophus affinis* as 2019-nCoV showed 96.2% of whole-genome identity to BatCoV RaTG13. The person-to-person transmission routes of 2019-nCoV included direct transmission, such as cough, sneeze, droplet inhalation transmission, and contact transmission, such as the contact with oral, nasal, and eye mucous membranes. 2019-nCoV can also be transmitted through the saliva, and the fetal–oral routes may also be a potential person-to-person transmission route. The participants in dental practice expose to tremendous risk of 2019-nCoV infection due to the face-to-face communication and the exposure to saliva, blood, and other body fluids, and the handling of sharp instruments. Dental professionals play great roles in preventing the transmission of 2019-nCoV. Here we recommend the infection control measures during dental practice to block the person-to-person transmission routes in dental clinics and hospitals.

## Introduction

An emergent pneumonia outbreak originated in Wuhan City, in the late December 2019^1^. The pneumonia infection has rapidly spread from Wuhan to most other provinces and other 24 countries^2,3^. World Health Organization declared a public health emergency of international concern over this global pneumonia outbreak on 30th January 2020.

The typical clinical symptoms of the patients who suffered from the novel viral pneumonia were fever, cough, and myalgia or fatigue with abnormal chest CT, and the less common symptoms were sputum production, headache, hemoptysis, and diarrhea^4–6^. This new infectious agent is more likely to affect older males to cause severe respiratory diseases^7,8^. Some of the clinical symptoms were different from the severe acute respiratory syndrome (SARS) caused by SARS coronavirus (SARS-CoV) that happened in 2002–2003, indicating that a new person-to-person transmission infectious agent has caused this emergent viral pneumonia outbreak^8,9^. Chinese researchers have quickly isolated a new virus from the patient and sequenced its genome (29,903 nucleotides)^10^. The infectious agent of this viral pneumonia happenening in Wuhan was finally identified as a novel coronavirus (2019-nCOV), the seventh member of the family of coronaviruses that infect humans^11^. On 11th February 2020, WHO named the novel viral pneumonia as “Corona Virus Disease (COVID19)”, while the international Committee on Taxonomy of Viruses (ICTV) suggested this novel coronavirus name as “SARS-CoV-2” due to the phylogenetic and taxonomic analysis of this novel coronavirus^12^.

## Characteristics of 2019 novel coronavirus

Coronaviruses belong to the family of *Coronaviridae*, of the order *Nidovirales*, comprising large, single, plus-stranded RNA as their genome^13,14^. Currently, there are four genera of coronaviruses: α-CoV, β-CoV, γ-CoV, and δ-CoV^15,16^. Most of the coronavirus can cause the infectious diseases in human and vertebrates. The α-CoV and β-CoV mainly infect the respiratory, gastrointestinal, and central nervous system of humans and mammals, while γ-CoV and δ-CoV mainly infect the birds^13,17–19^.

Usually, several members of the coronavirus cause mild respiratory disease in humans; however, SARS-CoV and the Middle East respiratory syndrome coronavirus (MERS-CoV) explored in 2002–2003 and in 2012, respectively, caused fatal severe respiratory diseases^20–22^. The SARS-CoV and MERS-CoV belong to the β-CoV^23,24^. 2019-nCoV explored in Wuhan also belongs to the β-CoV according to the phylogenetic analysis based on the viral genome^10,11^. Although the nucleotide sequence similarity is less than 80% between 2019-nCoV and SARS-CoV (about 79%) or MERS-CoV (about 50%), 2019-nCoV can also cause the fetal infection and spread more faster than the two other coronaviruses^7,9,11,25–27^. The genome nucleotide sequence identity between a coronavirus (BatCoV RaTG13) detected in the bat *Rhinolophus affinis* from Yunnan Province, China, and 2019-nCoV, was 96.2%, indicating that the natural host of 2019-nCoV may also be the *Rhinolophus affinis* bat^11^. However, the differences may also suggest that there is an or more intermediate hosts between the bat and human. A research team from the South China Agricultural University has invested more than 1 000 metagenomic samples from pangolins, and found that 70% pangolins contained β-CoV^28^. One of the coronaviruses they isolated from the pangolins comprised a genome that was very similar with that from 2019-nCoV, and the genome sequence similarity was 99%, indicating that the pangolin may be the intermediate host of 2019-nCoV^29^.

2019-nCoV possessed the typical coronavirus structure with the “spike protein” in the membrane envelope^30^, and also expressed other polyproteins, nucleoproteins, and membrane proteins, such as RNA polymerase, 3-chymotrypsin-like protease, papain-like protease, helicase, glycoprotein, and accessory proteins^10,11,30^. The S protein from coronavirus can bind to the receptors of the host to facilitate viral entry into target cells^31,32^. Although there are four amino acid variations of S protein between 2019-nCoV and SARS-CoV, 2019-nCoV can also bind to the human angiotensin-converting enzyme 2 (ACE2), the same host receptor for SARS-CoV, as 2019-nCoV can bind to the ACE2 receptor from the cells from human, bat, civet cat, and pig, but it cannot bind to the cells without ACE2^11,33–35^. A recombinant ACE2-Ig antibody, a SARS-CoV-specific human monoclonal antibody, and the serum from a convalescent SARS-CoV-infected patient, which can neutralize 2019-nCoV, confirmed ACE2 as the host receptor for 2019-nCoV^36–39^. The high affinity between ACE2 and 2019-nCoV S protein also suggested that the population with higher expression of ACE2 might be more susceptible to 2019-nCoV^40,41^. The cellular serine protease TMPRSS2 also contributed to the S-protein priming of 2019-nCoV, indicating that the TMPRSS2 inhibitor might constitute a treatment option^36^.

## The possible transmission routes of 2019-nCoV

The common transmission routes of novel coronavirus include direct transmission (cough, sneeze, and droplet inhalation transmission) and contact transmission (contact with oral, nasal, and eye mucous membranes)^42^. Although common clinical manifestations of novel coronavirus infection do not include eye symptoms, the analysis of conjunctival samples from confirmed and suspected cases of 2019-nCoV suggests that the transmission of 2019-nCoV is not limited to the respiratory tract^4^, and that eye exposure may provide an effective way for the virus to enter the body^43^.

In addition, studies have shown that respiratory viruses can be transmitted from person to person through direct or indirect contact, or through coarse or small droplets, and 2019-nCoV can also be transmitted directly or indirectly through saliva^44^. Notably, a report of one case of 2019-nCoV infection in Germany indicates that transmission of the virus may also occur through contact with asymptomatic patients^45^.

Studies have suggested that 2019-nCoV may be airborne through aerosols formed during medical procedures^46^. It is notable that 2019-nCoV RNA could also be detected by rRT-PCR testing in a stool specimen collected on day 7 of the patient’s illness^47^. However, the aerosol transmission route and the fecal–oral transmission route concerned by the public still need to be further studied and confirmed.

## Possible transmission routes of 2019-nCoV in dental clinics

Since 2019-nCoV can be passed directly from person to person by respiratory droplets, emerging evidence suggested that it may also be transmitted through contact and fomites^43,48^. In addition, the asymptomatic incubation period for individuals infected with 2019-nCov has been reported to be ~1–14 days, and after 24 days individuals were reported, and it was confirmed that those without symptoms can spread the virus^4,5,49^. To et al. reported that live viruses were present in the saliva of infected individuals by viral culture method^43^. Furthermore, it has been confirmed that 2019-nCov enters the cell in the same path as SARS coronavirus, that is, through the ACE2 cell receptor^25^. 2019-nCoV can effectively use ACE2 as a receptor to invade cells, which may promote human-to-human transmission^11^. ACE2^+^ cells were found to be abundantly present throughout the respiratory tract, as well as the cells morphologically compatible with salivary gland duct epithelium in human mouth. ACE2^+^ epithelial cells of salivary gland ducts were demonstrated to be a class early targets of SARS-CoV infection^50^, and 2019-nCoV is likely to be the same situation, although no research has been reported so far.

**Fig. 1:**
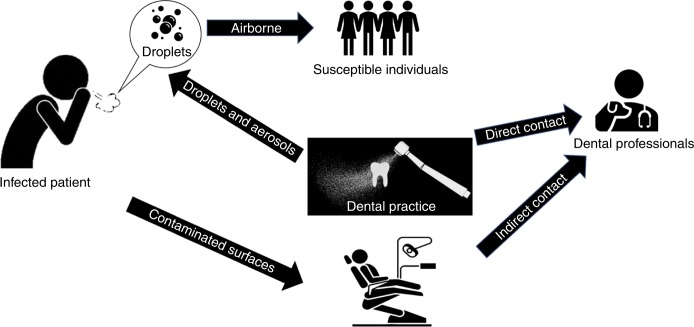
Illustration of transmission routes of 2019-nCoV in dental clinics and hospitals

Dental patients and professionals can be exposed to pathogenic microorganisms, including viruses and bacteria that infect the oral cavity and respiratory tract. Dental care settings invariably carry the risk of 2019-nCoV infection due to the specificity of its procedures, which involves face-to-face communication with patients, and frequent exposure to saliva, blood, and other body fluids, and the handling of sharp instruments. The pathogenic microorganisms can be transmitted in dental settings through inhalation of airborne microorganisms that can remain suspended in the air for long periods^51^, direct contact with blood, oral fluids, or other patient materials^52^, contact of conjunctival, nasal, or oral mucosa with droplets and aerosols containing microorganisms generated from an infected individual and propelled a short distance by coughing and talking without a mask^53,54^, and indirect contact with contaminated instruments and/or environmental surfaces^50^. Infections could be present through any of these conditions involved in an infected individual in dental clinics and hospitals, especially during the outbreak of 2019-nCoV (Fig. 1).

### Airborne spread

The airborne spread of SARS-Cov (severe acute respiratory syndrome coronavirus) is well-reported in many literatures. The dental papers show that many dental procedures produce aerosols and droplets that are contaminated with virus^55^. Thus, droplet and aerosol transmission of 2019-nCoV are the most important concerns in dental clinics and hospitals, because it is hard to avoid the generation of large amounts of aerosol and droplet mixed with patient’s saliva and even blood during dental practice^53^. In addition to the infected patient’s cough and breathing, dental devices such as high-speed dental handpiece uses high-speed gas to drive the turbine to rotate at high speed and work with running water. When dental devices work in the patient’s oral cavity, a large amount of aerosol and droplets mixed with the patient’s saliva or even blood will be generated. Particles of droplets and aerosols are small enough to stay airborne for an extended period before they settle on environmental surfaces or enter the respiratory tract. Thus, the 2019-nCoV has the potential to spread through droplets and aerosols from infected individuals in dental clinics and hospitals.

### Contact spread

A dental professional’s frequent direct or indirect contact with human fluids, patient materials, and contaminated dental instruments or environmental surfaces makes a possible route to the spread of viruses^53^. In addition, dental professionals and other patients have likely contact of conjunctival, nasal, or oral mucosa with droplets and aerosols containing microorganisms generated from an infected individual and propelled a short distance by coughing and talking without a mask. Effective infection control strategies are needed to prevent the spread of 2019-nCoV through these contact routines.

### Contaminated surfaces spread

Human coronaviruses such as SARS-CoV, Middle East Respiratory Syndrome coronavirus (MERS-CoV), or endemic human coronaviruses (HCoV) can persist on surfaces like metal, glass, or plastic for up to a couple of days^51,56^. Therefore, contaminated surfaces that are frequently contacted in healthcare settings are a potential source of coronavirus transmission. Dental practices derived droplets and aerosols from infected patients, which likely contaminate the whole surface in dental offices. In addition, it was shown at room temperature that HCoV remains infectious from 2 h up to 9 days, and persists better at 50% compared with 30% relative humidity. Thus, keeping a clean and dry environment in the dental office would help decrease the persistence of 2019-nCoV.

## Infection controls for dental practice

Dental professionals should be familiar with how 2019-nCoV is spread, how to identify patients with 2019-nCoV infection, and what extra-protective measures should be adopted during the practice, in order to prevent the transmission of 2019-nCoV. Here we recommend the infection control measures that should be followed by dental professionals, particularly considering the fact that aerosols and droplets were considered as the main spread routes of 2019-nCoV. Our recommendations are based on *the Guideline for the Diagnosis and Treatment of Novel Coronavirus Pneumonia* (the 5th edition) (http://www.nhc.gov.cn/yzygj/s7653p/202002/3b09b894ac9b4204a79db5b8912d4440.shtml), *the Guideline for the Prevention and Control of Novel Coronavirus Pneumonia in Medical Institutes* (the 1st edition) (http://www.nhc.gov.cn/yzygj/s7659/202001/b91fdab7c304431eb082d67847d27e14.shtml), and *the Guideline for the Use of Medical Protective Equipment in the Prevention and Control of Novel Coronavirus Pneumonia* (http://www.nhc.gov.cn/yzygj/s7659/202001/e71c5de925a64eafbe1ce790debab5c6.shtml) released by the National Health Commission of the People’s Republic of China, and the practice experience in West China Hospital of Stomatology related to the outbreak of 2019-nCoV transmission.

### Patient evaluation

First of all, dental professionals should be able to identify a suspected case of COVID-19. To date that this paper was drafted, the National Health Commission of the People’s Republic of China has released the 5th edition of the *Guideline for the Diagnosis and Treatment of Novel Coronavirus Pneumonia*. In general, a patient with COVID-19 who is in the acute febrile phase of the disease is not recommended to visit the dental clinic. If this does occur, the dental professional should be able to identify the patient with suspected 2019-nCoV infection, and should not treat the patient in the dental clinic, but immediately quarantine the patient and report to the infection control department as soon as possible, particularly in the epidemic period of 2019-nCoV.

The body temperature of the patient should be measured in the first place. A contact-free forehead thermometer is strongly recommended for the screening. A questionnaire should be used to screen patients with potential infection of 2019-nCoV before they could be led to the dental chair-side. These questions should include the following: (1) Do you have fever or experience fever within the past 14 days? (2) Have you experienced a recent onset of respiratory problems, such as a cough or difficulty in breathing within the past 14 days? (3) Have you, within the past 14 days, traveled to Wuhan city and its surrounding areas, or visited the neighborhood with documented 2019-nCoV transmission? (4) Have you come into contact with a patient with confirmed 2019-nCoV infection within the past 14 days? (5) Have you come into contact with people who come from Wuhan city and its surrounding areas, or people from the neighborhood with recent documented fever or respiratory problems within the past 14 days? (6) Are there at least two people with documented experience of fever or respiratory problems within the last 14 days having close contact with you? (7) Have you recently participated in any gathering, meetings, or had close contact with many unacquainted people?

If a patient replies “yes” to any of the screening questions, and his/her body temperature is below 37.3 °C, the dentist can defer the treatment until 14 days after the exposure event. The patient should be instructed to self-quarantine at home and report any fever experience or flu-like syndrome to the local health department. If a patient replies “yes” to any of the screening questions, and his/her body temperature is no less than 37.3 °C, the patient should be immediately quarantined, and the dental professionals should report to the infection control department of the hospital or the local health department. If a patient replies “no” to all the screening questions, and his/her body temperature is below 37.3 °C, the dentist can treat the patient with extra- protection measures, and avoids spatter or aerosol-generating procedures to the best. If a patient replies “no” to all the screening questions, but his/her body temperature is no less than 37.3 °C, the patient should be instructed to the fever clinics or special clinics for COVID-19 for further medical care.

### Hand hygiene

Fecal–oral transmission has been reported for 2019-nCoV, which underlines the importance of hand hygiene for dental practice. Although appropriate hand hygiene is the routine prerequisite for dental practice, hand-washing compliance is relatively low, which imposes a great challenge to the infection control during the epidemic period of 2019-nCoV transmission. Reinforcement for good hand hygiene is of the utmost importance. A two-before-and-three-after hand hygiene guideline is proposed by the infection control department of the West China Hospital of Stomatology, Sichuan University, to reinforce the compliance of hand washing. Specifically, the oral professionals should wash their hands before patient examination, before dental procedures, after touching the patient, after touching the surroundings and equipment without disinfection, and after touching the oral mucosa, damaged skin or wound, blood, body fluid, secretion, and excreta. More caution should be taken for the dental professionals to avoid touching their own eyes, mouth, and nose.

### Personal protective measures for the dental professionals

At present, there is no specific guideline for the protection of dental professionals from 2019-nCoV infection in the dental clinics and hospitals. Although no dental professional has been reported to acquire 2019-nCoV infection to the date the paper was drafted, the last experience with the SARS coronavirus has shown vast numbers of acquired infection of medical professionals in hospital settings^57^. Since airborne droplet transmission of infection is considered as the main route of spread, particularly in dental clinics and hospitals, barrier-protection equipment, including protective eyewear, masks, gloves, caps, face shields, and protective outwear, is strongly recommended for all healthcare givers in the clinic/hospital settings during the epidemic period of 2019-nCoV.

Based on the possibility of the spread of 2019-nCoV infection, three-level protective measures of the dental professionals are recommended for specific situations. (1) Primary protection (standard protection for staff in clinical settings). Wearing disposable working cap, disposable surgical mask, and working clothes (white coat), using protective goggles or face shield, and disposable latex gloves or nitrile gloves if necessary. (2) Secondary protection (advanced protection for dental professionals). Wearing disposable doctor cap, disposable surgical mask, protective goggles, face shield, and working clothes (white coat) with disposable isolation clothing or surgical clothes outside, and disposable latex gloves. (3) Tertiary protection (strengthened protection when contact patient with suspected or confirmed 2019-nCoV infection). Although a patient with 2019-nCoV infection is not expected to be treated in the dental clinic, in the unlikely event that this does occur, and the dental professional cannot avoid close contact, special protective outwear is needed. If protective outwear is not available, working clothes (white coat) with extra disposable protective clothing outside should be worn. In addition, disposable doctor cap, protective goggles, face shield, disposable surgical mask, disposable latex gloves, and impermeable shoe cover should be worn.

### Mouthrinse before dental procedures

A preoperational antimicrobial mouthrinse is generally believed to reduce the number of oral microbes. However, as instructed by the *Guideline for the Diagnosis and Treatment of Novel Coronavirus Pneumonia* (the 5th edition) released by the National Health Commission of the People’s Republic of China, chlorhexidine, which is commonly used as mouthrinse in dental practice, may not be effective to kill 2019-nCoV. Since 2019-nCoV is vulnerable to oxidation, preprocedural mouthrinse containing oxidative agents such as 1% hydrogen peroxide or 0.2% povidone is recommended, for the purpose of reducing the salivary load of oral microbes, including potential 2019-nCoV carriage. A preprocedural mouthrinse would be most useful in cases when rubber dam cannot be used.

### Rubber dam isolation

The use of rubber dams can significantly minimize the production of saliva- and blood-contaminated aerosol or spatter, particularly in cases when high-speed handpieces and dental ultrasonic devices are used. It has been reported that the use of rubber dam could significantly reduce airborne particles in ~3-foot diameter of the operational field by 70%^58^. When rubber dam is applied, extra high-volume suction for aerosol and spatter should be used during the procedures along with regular suction^59^. In this case, the implementation of a complete four-hand operation is also necessary. If rubber dam isolation is not possible in some cases, manual devices, such as Carisolv and hand scaler, are recommended for caries removal and periodontal scaling, in order to minimize the generation of aerosol as much as possible.

### Anti-retraction handpiece

The high-speed dental handpiece without anti-retraction valves may aspirate and expel the debris and fluids during the dental procedures. More importantly, the microbes, including bacteria and virus, may further contaminate the air and water tubes within the dental unit, and thus can potentially cause cross-infection. Our study has shown that the anti-retraction high-speed dental handpiece can significantly reduce the backflow of oral bacteria and HBV into the tubes of the handpiece and dental unit as compared with the handpiece without anti-retraction function^60^. Therefore, the use of dental handpieces without anti-retraction function should be prohibited during the epidemic period of COVID-19. Anti-retraction dental handpiece with specially designed anti-retractive valves or other anti-reflux designs are strongly recommended as an extra preventive measure for cross-infection^59^. Therefore, the use of dental handpieces without anti-retraction function should be prohibited during the epidemic period of COVID-19. Anti-retraction dental handpiece with specially designed anti-retractive valves or other anti-reflux designs are strongly recommended as an extra preventive measure for cross-infection.

### Disinfection of the clinic settings

Medical institutions should take effective and strict disinfection measures in both clinic settings and public area. The clinic settings should be cleaned and disinfected in accordance with *the Protocol for the Management of Surface Cleaning and Disinfection of Medical Environment (WS/T 512-2016)* released by the National Health Commission of the People’s Republic of China. Public areas and appliances should also be frequently cleaned and disinfected, including door handles, chairs, and desks. The elevator should be disinfected regularly. People taking elevators should wear masks correctly and avoid direct contact with buttons and other objects.

### Management of medical waste

The medical waste (including disposable protective equipment after use) should be transported to the temporary storage area of the medical institute timely. The reusable instrument and items should be pretreated, cleaned, sterilized, and properly stored in accordance with *the Protocol for the Disinfection and Sterilization of Dental Instrument (WS 506-2016)* released by the National Health Commission of the People’s Republic of China. The medical and domestic waste generated by the treatment of patients with suspected or confirmed 2019-nCoV infection are regarded as infectious medical waste. Double-layer yellow color medical waste package bags and “gooseneck” ligation should be used. The surface of the package bags should be marked and disposed according to the requirement for the management of medical waste.

## Summary

Since December 2019, the newly discovered coronavirus (2019-nCov) has caused the outbreak of pneumonia in Wuhan and throughout China. 2019-nCov enters host cells through human cell receptor ACE2, the same with SARS-CoV, but with higher binding affinity^61^. The rapidly increasing number of cases and evidence of human-to-human transmission suggested that the virus was more contagious than SARS-CoV and MERS-CoV^9,25,27,61^. By mid-February 2020, a large number of infections of medical staff have been reported^62^, and the specific reasons for the failure of protection need to be further investigated. Although clinics such as stomatology have been closed during the epidemic, a large number of emergency patients still go to the dental clinics and hospitals for treatment. We have summarized the possible transmission routes of 2019-nCov in stomatology, such as the airborne spread, contact spread, and contaminated surface spread. We also reviewed several detailed practical strategies to block virus transmission to provide a reference for preventing the transmission of 2019-nCov during dental diagnosis and treatment, including patient evaluation, hand hygiene, personal protective measures for the dental professionals, mouthrinse before dental procedures, rubber dam isolation, anti-retraction handpiece, disinfection of the clinic settings, and management of medical waste.
